# Shall We Use the Uterine Sound to Correct Backward Displacements of the Uterus?

**Published:** 1891-11

**Authors:** Clinton Cushing

**Affiliations:** San Francisco, Cal.; Professor of Gynecology, Cooper Medical College; 636 Sutter Street


### SHALL WE USE THE UTERINE SOUND TO CORRECT BACKWARD DISPLACEMENTS OF THE UTERUS?[1]

By CLINTON CUSHING, M. D., San Francisco, Cal.
Professor of Gynecology, Cooper Medical College.

It is almost certain that this question will meet very opposite opinions from the members of this Association. Now, as opposition is the very life of, and the cause of progress in such an organization, it is to be hoped that a fair amouut of consideration will be accorded this subject. The profession seems divided into three parties. A few who, under no circumstances, would introduce a sound into the uterus to correct a displacement; a large number who would do so under certain circumstances, and a small number who would recklessly do so in almost any case. The object of this communication is to discuss the conditions when we should or should not use the sound.

As all the older members know, gynecology was very poorly, if at all, taught in our medical schools here, or in any part of the world, twenty-five years ago. As a separate branch of practice or study it had scarcely an existence. Therefore, when I received my degree, I knew practically nothing about the diseases peculiar to women, and now began a blind groping after knowledge, at the expense of much anxiety and worry, and at the cost of much unnecessary pain and suffering to the unfortunate women who fell into my hands. Pelvic peritonitis, pelvic abscess, and death followed promptly in a considerable number of cases. The various methods pursued, to relieve the patients of the symptoms of which they complained, were crude. The merits of cleanliness were unknown. My armamentarium consisted of a Fergusson speculum, and a piece of nitrate of silver fastened in a goose-quill. The principal disease to be treated was the so-called ulceration of the cervix. The knowledge of pelvic inflammation and abscess, of tubal disease and extra-uterine pregnancy, possessed by the average practitioner of medicine, was insufficient to be of service in the management of his cases. Little wonder, then, that mistakes and blunders were frequent and disastrous. The intelligent graduate of today can scarcely appreciate the difficulties under which we labored thirty years ago. Happy the gynecologist who has had the road smoothed and made ready for him by the labors and painful experiences of others!

---

1. Read at the Fourth Annual Meeting of the American Association of Obstetricians and Gynecologists in New York, September, 1891.

A problem presented itself at the outset of my practice which grew in importance continually: Why did a simple treatment of an erosion of the cervix or a cervical endometritis, set up a dangerous pelvic inflammation in one case, when in twenty other apparently similar cases the treatment was not harmful? It has been the good fortune of all of us to have the truth of this question settled during the past few years, largely through the labors and energy of Mr. Lawson Tait. He demonstrated, by many abdominal sections, that pyosalpinx was the common concomitant of acute or chronic pelvic inflammation; this truth has been verified since by many able men in all parts of the world, and the conclusion has been generally arrived, at that the escape of pus or muco-purulent matter from the Fallopian tube into the abdominal cavity is the common cause of pelvic peritonitis.

This point being conceded, I will now proceed to set forth the results of my observations in the use of the uterine sound in replacing retroverted and retroflexed uteri. I will cite a case that, I doubt not, will be recognized as an old acquaintance by all present.

A woman applies for advice concerning the usual symptoms of pelvic disease,—backache, pain in region of uterus and ovaries, inability to stand and walk without increased suffering, with the various reflex symptoms of stomach and head in varying degrees. She gives a history of some acute pelvic inflammation, or not, as the case may be. Upon examination, a marked retroversion of the uterus is found. Perhaps a prolapse of one or both ovaries. The uterus is quite firmly fixed. The tissues about the uterus are not very tender, and the previous history is somewhat obscure.

First, an effort is made by manual and bi-manual pressure to correct the displacement. This failing, the woman is placed upon her elbows and knees, a Sims speculum is introduced and held by an assistant, and now, the cervix being well drawn down by a tenaculum, an effort is made with a firm ball of cotton held in a dressing-forceps to push the body of the uterus forward, the force being applied from the region of Douglas's pouch. If the effort is not successful in loosening the uterus, a firm tampon is applied behind the cervix, and the vagina is filled with cotton, so as to crowd the uterus well up in the pelvis. This procedure is repeated two or three times a week, but without any good result. The troublesome symptoms are not relieved. I now take a stiff copper sound, or, what is better, a number eight Otis's steel urethral sound, and, introducing it while the woman is in the knee-elbow position, with the cavity backward, describe an arc with the handle of the sound, thus bringing the uterus into a condition of anteversion, with the least possible violence to the uterine mucous membrane.

By this procedure is at once made manifest the firmness of the adhesions between the posterior surface of the uterus and the adjacent structures. If the uterus can be placed in a state of anteversion by the use of a moderate amount of force thus applied, well and good. The effort is abandoned, for the time, if the pain be marked, or unusual force required. A vaginal tampon of glycerine and cotton is applied, and the patient ordered confined to bed for forty-eight hours. This procedure is repeated at the expiration of three or four days, unless inflammation supervenes. If it be found that the uterus cannot be placed in a state of anteversion without the using of great force, or if it be found that the adhesions to the rectum are so firm that when the sound is removed the uterus immediately springs back as though drawn by a rubber band, it is probable that the plan of correcting the displacement by the sound will prove a failure.

During the past ten years I have successfully treated a large number of cases similar to the one described above, have invariably used this method, and I have met but few cases where this plan has proved a failure. Now, an important question arises: How large a proportion of these women were made worse as a consequence of this manipulation? In other words, in how many did some pelvic inflammation supervene? Certainly in not more than one per cent. We are told by some writers that the introduction of any instrument into the cavity of the uterus is a very dangerous procedure. If this is true, why do I have an immunity? For the very simple reason that I have learned to draw the line regarding any kind of intraüterine instrumentation, where, from the history of the case, or from a very thorough and careful manual and bi-manual examination, there exists a reason for believing that there is disease of the Fallopian tubes.

The limits of this paper will not permit a discussion of the diagnosis of diseases of the Fallopian tubes, but when such a disease of the tube or tubes is clearly diagnosticated, then, and only then, in my opinion, is the use of the intraüterine sound contradicted in retroversion or retroflexion of the uterus.

Since the Fallopian tubes are a continuation of the uterine canal, and similar in structure, any irritating matter contained therein is liable to be forced outward into the peritoneal cavity, by any wave of contraction commencing in the uterus and extending to the tube, from whatever cause, whether it be the introduction of a sound, violent exercise, sudden pelvic congestion, or the sexual act. It is admitted that the objections of Emmet and his followers to the use of the sound are, to a certain extent, logical, but it is also claimed,—first, that those objections apply only to cases complicated by pus in the Fallopian tubes; and second, that the same objections apply with equal, if not greater force, to any other form of treatment in the same condition.

636 Sutter Street.

